# Ethyl 2-[(2-oxo-2*H*-chromen-7-yl)­oxy]acetate

**DOI:** 10.1107/S1600536813005539

**Published:** 2013-03-06

**Authors:** Hoong-Kun Fun, Ching Kheng Quah, Krishnendu Aich, Sangita Das, Shyamaprosad Goswami

**Affiliations:** a X-ray Crystallography Unit, School of Physics, Universiti Sains Malaysia, 11800 USM, Penang, Malaysia; b Department of Pharmaceutical Chemistry, College of Pharmacy, King Saud University, PO Box 2457, Riyadh 11451, Saudi Arabia; c Department of Chemistry, Bengal Engineering and Science University, Shibpur, Howrah 711 103, India

## Abstract

In the title compound, C_13_H_12_O_5_, the mean plane of the 2*H*-chromene ring system (r.m.s deviation = 0.026 Å) forms a dihedral angle of 81.71 (6)° with the mean plane of ethyl 2-hy­droxy­acetate moiety (r.m.s deviation = 0.034 Å). In the crystal, C—H⋯O hydrogen bonds result in the formation of zigzag layers parallel to the *bc* plane.

## Related literature

For general background to and the high emission quantum yield, photo stability and good solubility in common solvents of coumarin derivatives, see: Xie *et al.* (2012 ▶); Liu *et al.* (2012 ▶). For standard bond-length data, see: Allen *et al.* (1987 ▶). For the stability of the temperature controller used for the data collection, see: Cosier & Glazer (1986 ▶). For related structures, see: Arshad *et al.* (2010*a* ▶,*b* ▶).
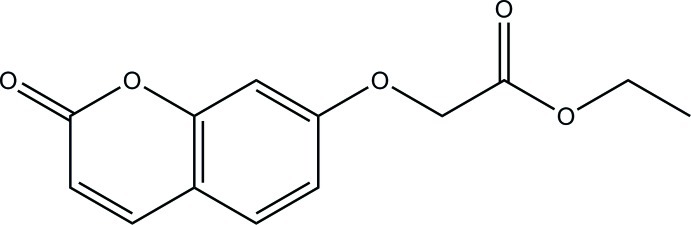


## Experimental

### 

#### Crystal data

C_13_H_12_O_5_
*M* *_r_* = 248.23
Monoclinic, 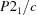

*a* = 8.1435 (2) Å
*b* = 16.4887 (4) Å
*c* = 10.5506 (3) Å
β = 125.882 (2)°
*V* = 1147.84 (5) Å^3^
*Z* = 4
Mo *K*α radiation
μ = 0.11 mm^−1^
*T* = 100 K
0.31 × 0.24 × 0.11 mm

#### Data collection

Bruker SMART APEXII CCD area-detector diffractometer
Absorption correction: multi-scan (*SADABS*; Bruker, 2009 ▶) *T*_min_ = 0.966, *T*_max_ = 0.988
12656 measured reflections
3344 independent reflections
2308 reflections with *I* > 2σ(*I*)
*R*_int_ = 0.035

#### Refinement

*R*[*F* ^2^ > 2σ(*F* ^2^)] = 0.049
*wR*(*F* ^2^) = 0.114
*S* = 1.04
3344 reflections
164 parameters
H-atom parameters constrained
Δρ_max_ = 0.32 e Å^−3^
Δρ_min_ = −0.24 e Å^−3^

### 

Data collection: *APEX2* (Bruker, 2009 ▶); cell refinement: *SAINT* (Bruker, 2009 ▶); data reduction: *SAINT*; program(s) used to solve structure: *SHELXTL* (Sheldrick, 2008 ▶); program(s) used to refine structure: *SHELXTL*; molecular graphics: *SHELXTL*; software used to prepare material for publication: *SHELXTL* and *PLATON* (Spek, 2009 ▶).

## Supplementary Material

Crystal structure: contains datablock(s) global, I. DOI: 10.1107/S1600536813005539/rz5046sup1.cif

Structure factors: contains datablock(s) I. DOI: 10.1107/S1600536813005539/rz5046Isup2.hkl

Supplementary material file. DOI: 10.1107/S1600536813005539/rz5046Isup3.cml

Additional supplementary materials:  crystallographic information; 3D view; checkCIF report

## Figures and Tables

**Table 1:** Hydrogen-bond geometry (Å, °)

*D*—H⋯*A*	*D*—H	H⋯*A*	*D*⋯*A*	*D*—H⋯*A*
C1—H1*A*⋯O2^i^	0.93	2.38	3.2913 (19)	166
C5—H5*A*⋯O5^ii^	0.93	2.55	3.373 (2)	147
C10—H10*B*⋯O2^i^	0.97	2.48	3.353 (2)	149

Symmetry codes: (i) 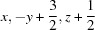; (ii) 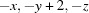.

**Fig. 1.**
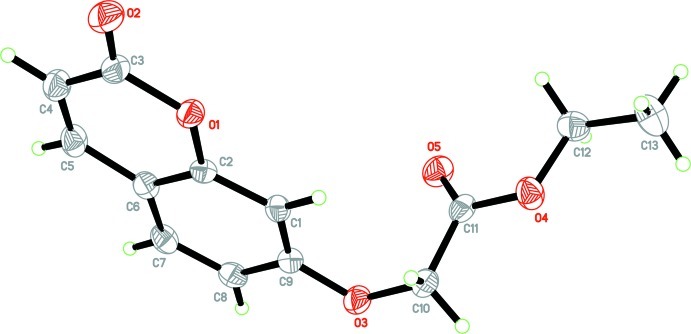
The molecular structure of the title compound showing 50% probability displacement ellipsoids for non-H atoms.

**Fig. 2.**
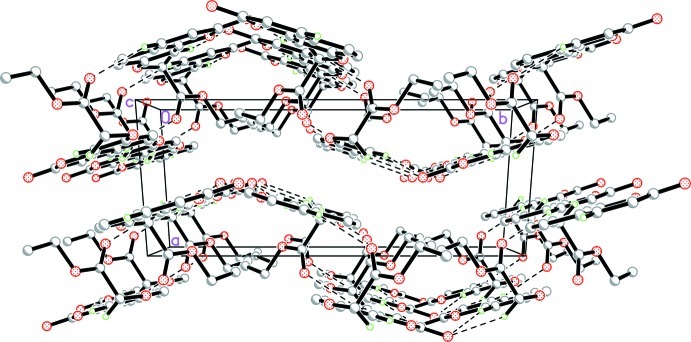
The crystal structure of the title compound, viewed along the c axis. H atoms not involved in hydrogen bonds (dashed lines) have been omitted for clarity.

## supplementary crystallographic information

### Comment

Coumarins are attractive fluorescent molecules due to their high emission quantum yield, photo stability and good solubility in common solvents. As a consequence of these features, coumarins are widely used as laser dyes (Xie *et al.*, 2012; Liu *et al.*, 2012). Herein, we report the crystal structure of ethyl 2-(2-oxo-2H-chromen-7-yloxy)acetate.

In the title molecule, Fig. 1, the mean plane of 2*H*-chromene ring system (O1/C1-C9, r.m.s deviation = 0.026 Å) forms a dihedral angle of 81.71 (6)° with the mean plane of ethyl 2-hydroxyacetate moiety (O1/N3/C9/C10, r.m.s deviation = 0.034 Å). Bond lengths (Allen *et al.*, 1987) and angles are within normal ranges and are comparable to those observed in related structures (Arshad *et al.*, 2010*a*, 2010*b*).

In the crystal structure (Fig. 2), molecules are linked *via* intermolecular C1–H1A···O2, C5–H5A···O5 and C10–H10B···O2 hydrogen bonds (Table 1) into zigzag layers parallel to the *bc* plane.

### Experimental

To a stirred solution of 7-hydroxycoumarin (500 mg, 3 mmol) in dry acetone, potassium carbonate (900 mg, 6 mmol) was added. After stirring for five minutes, ethyl chloroacetate (564 mg, 4.6 mmol) and a catalytic amount of TBAB were added to it. The whole reaction mixture was further stirred for 12 h at room temperature. After evaporation, water was added to it and the reaction mixture was extracted with chloroform thrice. The organic solvents were combined together and dried over anhydrous sodium sulphate and evaporated under reduced pressure. The crude product was purified through column chromatography (silica gel, 100-200 mesh size) using 15% ethyl acetate in petroleum ether as eluent to afford a pure colourless crystalline solid. Yield: 98%. M. p. 74-76 °C.

### Refinement

All H atoms were positioned geometrically and refined using a riding model with C–H = 0.93–0.97 Å and *U*_iso_(H) = 1.2 or 1.5 *U*_eq_(C). A rotating-group model was applied for the methyl group.

### Crystal data

**Table:**

C_13_H_12_O_5_	*F*(000) = 520
*M**_r_* = 248.23	*D*_x_ = 1.436 Mg m^−^^3^
Monoclinic, *P*2_1_/*c*	Mo *K*α radiation, λ = 0.71073 Å
Hall symbol: -P 2ybc	Cell parameters from 3372 reflections
*a* = 8.1435 (2) Å	θ = 2.5–29.4°
*b* = 16.4887 (4) Å	µ = 0.11 mm^−^^1^
*c* = 10.5506 (3) Å	*T* = 100 K
β = 125.882 (2)°	Block, colourless
*V* = 1147.84 (5) Å^3^	0.31 × 0.24 × 0.11 mm
*Z* = 4	

### Data collection

**Table:**

Bruker SMART APEXII CCD area-detector diffractometer	3344 independent reflections
Radiation source: fine-focus sealed tube	2308 reflections with *I* > 2σ(*I*)
Graphite monochromator	*R*_int_ = 0.035
φ and ω scans	θ_max_ = 30.0°, θ_min_ = 2.5°
Absorption correction: multi-scan (*SADABS*; Bruker, 2009)	*h* = −9→11
*T*_min_ = 0.966, *T*_max_ = 0.988	*k* = −23→15
12656 measured reflections	*l* = −14→14

### Refinement

**Table:**

Refinement on *F*^2^	Primary atom site location: structure-invariant direct methods
Least-squares matrix: full	Secondary atom site location: difference Fourier map
*R*[*F*^2^ > 2σ(*F*^2^)] = 0.049	Hydrogen site location: inferred from neighbouring sites
*wR*(*F*^2^) = 0.114	H-atom parameters constrained
*S* = 1.04	*w* = 1/[σ^2^(*F*_o_^2^) + (0.040*P*)^2^ + 0.4605*P*] where *P* = (*F*_o_^2^ + 2*F*_c_^2^)/3
3344 reflections	(Δ/σ)_max_ = 0.001
164 parameters	Δρ_max_ = 0.32 e Å^−^^3^
0 restraints	Δρ_min_ = −0.24 e Å^−^^3^

### Special details

**Table:**

Experimental. The crystal was placed in the cold stream of an Oxford Cryosystems Cobra open-flow nitrogen cryostat (Cosier & Glazer, 1986) operating at 100.0 (1) K.
Geometry. All esds (except the esd in the dihedral angle between two l.s. planes) are estimated using the full covariance matrix. The cell esds are taken into account individually in the estimation of esds in distances, angles and torsion angles; correlations between esds in cell parameters are only used when they are defined by crystal symmetry. An approximate (isotropic) treatment of cell esds is used for estimating esds involving l.s. planes.
Refinement. Refinement of F^2^ against ALL reflections. The weighted R-factor wR and goodness of fit S are based on F^2^, conventional R-factors R are based on F, with F set to zero for negative F^2^. The threshold expression of F^2^ > 2sigma(F^2^) is used only for calculating R-factors(gt) etc. and is not relevant to the choice of reflections for refinement. R-factors based on F^2^ are statistically about twice as large as those based on F, and R- factors based on ALL data will be even larger.

### Fractional atomic coordinates and isotropic or equivalent isotropic displacement parameters (Å^2^)

**Table:**

	*x*	*y*	*z*	*U*_iso_*/*U*_eq_	
O1	0.39589 (16)	0.81519 (6)	0.17293 (12)	0.0225 (2)	
O2	0.48206 (18)	0.71922 (7)	0.07680 (13)	0.0311 (3)	
O3	0.23054 (17)	1.01606 (6)	0.40644 (13)	0.0273 (3)	
O4	0.06112 (16)	0.85696 (7)	0.53551 (13)	0.0284 (3)	
O5	−0.09962 (17)	0.91734 (7)	0.30015 (13)	0.0305 (3)	
C1	0.3155 (2)	0.91211 (9)	0.29088 (17)	0.0211 (3)	
H1A	0.3389	0.8732	0.3635	0.025*	
C2	0.3385 (2)	0.89394 (9)	0.17359 (16)	0.0193 (3)	
C3	0.4238 (2)	0.78861 (10)	0.06252 (18)	0.0246 (3)	
C4	0.3788 (2)	0.84577 (10)	−0.05922 (18)	0.0268 (3)	
H4A	0.3890	0.8291	−0.1386	0.032*	
C5	0.3227 (2)	0.92215 (10)	−0.05837 (18)	0.0270 (3)	
H5A	0.2957	0.9579	−0.1367	0.032*	
C6	0.3039 (2)	0.94961 (9)	0.06176 (17)	0.0221 (3)	
C7	0.2475 (2)	1.02864 (10)	0.07243 (18)	0.0254 (3)	
H7A	0.2248	1.0677	0.0001	0.031*	
C8	0.2255 (2)	1.04899 (9)	0.18806 (18)	0.0243 (3)	
H8A	0.1900	1.1016	0.1947	0.029*	
C9	0.2567 (2)	0.98993 (9)	0.29621 (17)	0.0225 (3)	
C10	0.2416 (2)	0.95782 (10)	0.51046 (18)	0.0253 (3)	
H10A	0.2669	0.9853	0.6017	0.030*	
H10B	0.3541	0.9213	0.5454	0.030*	
C11	0.0474 (2)	0.90906 (9)	0.43309 (17)	0.0232 (3)	
C12	−0.1214 (2)	0.80927 (10)	0.4789 (2)	0.0301 (4)	
H12A	−0.1582	0.7776	0.3882	0.036*	
H12B	−0.2336	0.8449	0.4489	0.036*	
C13	−0.0765 (3)	0.75434 (11)	0.6084 (2)	0.0381 (4)	
H13A	−0.1945	0.7226	0.5743	0.057*	
H13B	−0.0400	0.7862	0.6975	0.057*	
H13C	0.0337	0.7190	0.6365	0.057*	

### Atomic displacement parameters (Å^2^)

**Table:**

	*U*^11^	*U*^22^	*U*^33^	*U*^12^	*U*^13^	*U*^23^
O1	0.0289 (6)	0.0198 (5)	0.0218 (5)	0.0002 (4)	0.0166 (5)	0.0001 (4)
O2	0.0396 (7)	0.0241 (6)	0.0342 (6)	−0.0001 (5)	0.0243 (6)	−0.0039 (5)
O3	0.0382 (6)	0.0211 (6)	0.0293 (6)	−0.0009 (5)	0.0236 (5)	−0.0021 (5)
O4	0.0249 (6)	0.0342 (6)	0.0231 (5)	−0.0025 (5)	0.0125 (5)	0.0046 (5)
O5	0.0309 (6)	0.0332 (7)	0.0220 (6)	0.0021 (5)	0.0125 (5)	0.0023 (5)
C1	0.0242 (7)	0.0208 (7)	0.0189 (7)	−0.0017 (6)	0.0129 (6)	0.0021 (6)
C2	0.0181 (7)	0.0183 (7)	0.0185 (7)	−0.0015 (5)	0.0091 (6)	−0.0009 (5)
C3	0.0237 (7)	0.0282 (9)	0.0227 (8)	−0.0039 (6)	0.0140 (7)	−0.0054 (6)
C4	0.0269 (8)	0.0334 (9)	0.0218 (8)	−0.0023 (7)	0.0152 (7)	−0.0011 (7)
C5	0.0268 (8)	0.0342 (9)	0.0203 (7)	−0.0013 (7)	0.0139 (7)	0.0047 (7)
C6	0.0191 (7)	0.0263 (8)	0.0183 (7)	−0.0021 (6)	0.0095 (6)	0.0021 (6)
C7	0.0237 (7)	0.0246 (8)	0.0255 (8)	−0.0001 (6)	0.0130 (7)	0.0067 (6)
C8	0.0242 (8)	0.0187 (8)	0.0285 (8)	−0.0008 (6)	0.0146 (7)	0.0006 (6)
C9	0.0232 (7)	0.0237 (8)	0.0216 (7)	−0.0036 (6)	0.0136 (6)	−0.0027 (6)
C10	0.0289 (8)	0.0270 (8)	0.0219 (7)	−0.0006 (7)	0.0160 (7)	−0.0016 (6)
C11	0.0280 (8)	0.0224 (8)	0.0213 (7)	0.0038 (6)	0.0156 (7)	0.0000 (6)
C12	0.0256 (8)	0.0314 (9)	0.0295 (8)	−0.0033 (7)	0.0142 (7)	−0.0025 (7)
C13	0.0324 (9)	0.0384 (10)	0.0474 (11)	0.0026 (8)	0.0257 (9)	0.0110 (9)

### Geometric parameters (Å, º)

**Table:**

O1—C2	1.3814 (17)	C5—H5A	0.9300
O1—C3	1.3827 (17)	C6—C7	1.408 (2)
O2—C3	1.2136 (19)	C7—C8	1.375 (2)
O3—C9	1.3692 (17)	C7—H7A	0.9300
O3—C10	1.4211 (18)	C8—C9	1.405 (2)
O4—C11	1.3328 (18)	C8—H8A	0.9300
O4—C12	1.4642 (19)	C10—C11	1.516 (2)
O5—C11	1.2045 (18)	C10—H10A	0.9700
C1—C9	1.382 (2)	C10—H10B	0.9700
C1—C2	1.389 (2)	C12—C13	1.495 (2)
C1—H1A	0.9300	C12—H12A	0.9700
C2—C6	1.387 (2)	C12—H12B	0.9700
C3—C4	1.456 (2)	C13—H13A	0.9600
C4—C5	1.341 (2)	C13—H13B	0.9600
C4—H4A	0.9300	C13—H13C	0.9600
C5—C6	1.436 (2)		
			
C2—O1—C3	121.70 (12)	C9—C8—H8A	120.1
C9—O3—C10	118.20 (12)	O3—C9—C1	123.83 (13)
C11—O4—C12	115.63 (12)	O3—C9—C8	115.29 (13)
C9—C1—C2	117.86 (13)	C1—C9—C8	120.85 (14)
C9—C1—H1A	121.1	O3—C10—C11	111.63 (12)
C2—C1—H1A	121.1	O3—C10—H10A	109.3
O1—C2—C6	121.23 (13)	C11—C10—H10A	109.3
O1—C2—C1	115.48 (12)	O3—C10—H10B	109.3
C6—C2—C1	123.28 (14)	C11—C10—H10B	109.3
O2—C3—O1	116.08 (14)	H10A—C10—H10B	108.0
O2—C3—C4	126.72 (14)	O5—C11—O4	124.81 (15)
O1—C3—C4	117.20 (14)	O5—C11—C10	125.30 (14)
C5—C4—C3	120.84 (14)	O4—C11—C10	109.86 (12)
C5—C4—H4A	119.6	O4—C12—C13	107.85 (13)
C3—C4—H4A	119.6	O4—C12—H12A	110.1
C4—C5—C6	120.98 (14)	C13—C12—H12A	110.1
C4—C5—H5A	119.5	O4—C12—H12B	110.1
C6—C5—H5A	119.5	C13—C12—H12B	110.1
C2—C6—C7	117.25 (14)	H12A—C12—H12B	108.5
C2—C6—C5	117.92 (14)	C12—C13—H13A	109.5
C7—C6—C5	124.81 (14)	C12—C13—H13B	109.5
C8—C7—C6	121.00 (14)	H13A—C13—H13B	109.5
C8—C7—H7A	119.5	C12—C13—H13C	109.5
C6—C7—H7A	119.5	H13A—C13—H13C	109.5
C7—C8—C9	119.71 (14)	H13B—C13—H13C	109.5
C7—C8—H8A	120.1		
			
C3—O1—C2—C6	0.8 (2)	C2—C6—C7—C8	−0.8 (2)
C3—O1—C2—C1	179.82 (13)	C5—C6—C7—C8	177.46 (15)
C9—C1—C2—O1	−179.53 (12)	C6—C7—C8—C9	−0.9 (2)
C9—C1—C2—C6	−0.6 (2)	C10—O3—C9—C1	−7.5 (2)
C2—O1—C3—O2	177.16 (13)	C10—O3—C9—C8	174.34 (13)
C2—O1—C3—C4	−3.51 (19)	C2—C1—C9—O3	−179.32 (13)
O2—C3—C4—C5	−177.40 (16)	C2—C1—C9—C8	−1.3 (2)
O1—C3—C4—C5	3.4 (2)	C7—C8—C9—O3	−179.80 (13)
C3—C4—C5—C6	−0.5 (2)	C7—C8—C9—C1	2.0 (2)
O1—C2—C6—C7	−179.50 (13)	C9—O3—C10—C11	−78.32 (16)
C1—C2—C6—C7	1.6 (2)	C12—O4—C11—O5	−1.9 (2)
O1—C2—C6—C5	2.1 (2)	C12—O4—C11—C10	176.37 (12)
C1—C2—C6—C5	−176.81 (14)	O3—C10—C11—O5	0.2 (2)
C4—C5—C6—C2	−2.2 (2)	O3—C10—C11—O4	−178.06 (12)
C4—C5—C6—C7	179.51 (15)	C11—O4—C12—C13	179.49 (14)

### Hydrogen-bond geometry (Å, º)

**Table:**

*D*—H···*A*	*D*—H	H···*A*	*D*···*A*	*D*—H···*A*
C1—H1*A*···O2^i^	0.93	2.38	3.2913 (19)	166
C5—H5*A*···O5^ii^	0.93	2.55	3.373 (2)	147
C10—H10*B*···O2^i^	0.97	2.48	3.353 (2)	149

Symmetry codes: (i) *x*, −*y*+3/2, *z*+1/2; (ii) −*x*, −*y*+2, −*z*.
